# Inter-Plant Vibrational Communication in a Leafhopper Insect

**DOI:** 10.1371/journal.pone.0019692

**Published:** 2011-05-05

**Authors:** Anna Eriksson, Gianfranco Anfora, Andrea Lucchi, Meta Virant-Doberlet, Valerio Mazzoni

**Affiliations:** 1 Research and Innovation Centre, Fondazione Edmund Mach, San Michele all'Adige, Italy; 2 Department of Coltivazione e Difesa delle Specie Legnose, University of Pisa, Pisa, Italy; 3 Department of Entomology, National Institute of Biology, Ljubljana, Slovenia; University of Arizona, United States of America

## Abstract

Vibrational communication is one of the least understood channels of communication. Most studies have focused on the role of substrate-borne signals in insect mating behavior, where a male and a female establish a stereotyped duet that enables partner recognition and localization. While the effective communication range of substrate-borne signals may be up to several meters, it is generally accepted that insect vibrational communication is limited to a continuous substrate. Until now, interplant communication in absence of physical contact between plants has never been demonstrated in a vibrational communicating insect. With a laser vibrometer we investigated transmission of natural and played back vibrational signals of a grapevine leafhopper, *Scaphoideus titanus*, when being transmitted between leaves of different cuttings without physical contact. Partners established a vibrational duet up to 6 cm gap width between leaves. Ablation of the antennae showed that antennal mechanoreceptors are not essential in detection of mating signals. Our results demonstrate for the first time that substrate discontinuity does not impose a limitation on communication range of vibrational signals. We also suggest that the behavioral response may depend on the signal intensity.

## Introduction

Substrate-borne vibrational signaling is a widespread form of animal communication, not only in arthropods [1], [2] but also among vertebrates [3], [4]. Although it has been recognized for centuries, its importance has long been overlooked [1], [2], [5]. As with any communication channel, the effective communication range of vibrational signals depends on the amplitude of the emitted signals, on attenuation and degradation during propagation [6]–[8] and on the sensitivity of the receiver's receptors [9]. Depending on the size, the communication range of vibrational signals can extend up to eight meters [6], [10]–[13]. At any rate, it is generally assumed to be limited to one plant or neighboring plants with interconnected roots or touching leaves [2], [10], [14], [15].

Until recently most studies on vibrational communication have been made within the range of few centimeters and have primarily focused on the species-specific vibrational repertoire (reviewed in [10], [16]). The ability of conspecifics to recognize and locate each other in the environment depends on the efficacy of their communication. In particular, species-specific signals used in sexual communication enable identification of the sender (species and sex) and provide information necessary to determine its location [17], [18]. In order to efficiently localize a conspecific partner, receivers should, in principle, determine not only a direction of the signal source, but also estimate its distance and adjust searching strategy accordingly. Currently there is no evidence of determination of source distance in plant-dwelling insects [19]. However, it has been hypothesized that on plants, insects may be able to roughly estimate the distance by the extent of distortion and degradation due to differences in attenuation and filtering of different frequency components in the signal [6].

Signals that are perceived by insects as substrate-borne vibrations usually have a low intensity air-borne component [10], [20], [21] that potentially may be detected over few centimeters by antennal receptors (e.g. [22]) or even by vibration receptors in the legs [23]. Antennal receptors suggested to be involved in perception of air-borne and substrate-borne vibrations have been described in *Oncopsis flavicollis* [24]–[25], *Nezara viridula* [26], and *Hyalesthes obsoletus* [27]. Therefore, we investigated whether continuity of the substrate is essential in the transmission of vibrational signals for successful communication between sexes.

As a model species we chose the leafhopper *Scaphoideus titanus* Ball (Hemiptera: Cicadellidae), a major pest of grapevine, that transmits the phytoplasma responsible of the grapevine yellow disease “Flavescence dorée” in Europe [28]. The role of vibrational signals in intraspecific communication and pair formation of *S. titanus* on a single grapevine leaf has been described in detail. Pair formation begins with a spontaneous emission of a male calling signal (MCS) which in response to female reply may extend into a courtship phrase (MCrP). Females don't emit vibrational signals spontaneously [29]. In absence of female reply males may perform the “call-fly” behavior [30], by alternating emissions of MCS with jumps from the plant [29].

We show here that discontinuity of substrate is not a barrier for communication in a vibrational communicating insect and that antennal receptors are not essential for detecting mating signals when partners are placed on discontinuous substrates. The results are discussed with regard to mate searching behavior associated with different levels of signal intensity.

## Results

### Test 1. Male-female inter-plant communication

**Figure 1:**
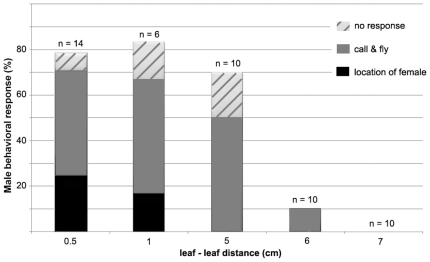
Male-female communication in *Scaphoideus titanus* recorded on leaves without direct contact (Test 1). Distances between upper and lower leaf were from 0.5 cm to 7 cm. The percentage of females that responded to the male calling signal (total column height) is divided according to the subsequent male behavioral response: mating duet, followed either by female location (black) or by call-fly (gray), and no male reaction (striped). n indicates the number of insect pairs tested.

We placed *S. titanus* male and female on different grapevine leaves separated by a gap of varying widths. In all trials males initiated communication behavior with emission of MCS and females were observed to reply to male calls up to a 6-cm gap distance (Figure 1). As a result of female responses, most males established a duet with the female that ended either with female location or “call-fly” behavior. Few males did not show any reaction to female responses. When mating duets were observed, they were composed of short series of male pulses alternated with one or more female pulses. Within the 5-cm gap distance, most females replied to male calls, although mate locations - achieved by the short jump from the upper leaf to the lower one with the female - were observed only at shorter distance. At 7-cm distance between leaves, none of the females responded to MCS.

### Test 2. Signal transmission

**Figure 2:**
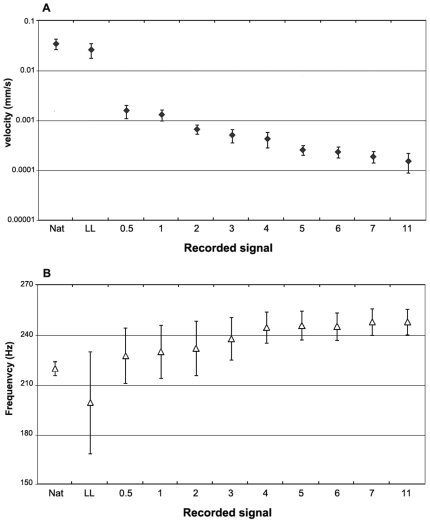
Signal properties measured on leaves with discontinuous substrate (Test 2). Mean (±SE) values of maximum substrate vibration velocity (mm/s) (A, logarithmic scale) and frequency (Hz) (B) of pulses from MCS (Male calling signal) are shown. While substrate velocity progressively decreased (Jonckheere test: J_0_ = 5.93, P<0.001) with the distance between leaves, the frequency increased (J_0_ = 2.29, P = 0.011). Nat: MCS emitted by natural male recorded on the same leaf; LL: MCS emitted by playback recorded on the same leaf; 0.5–11: MCS emitted on the lower leaf and recorded from the upper leaf with a progressive gap width of 0.5–11 cm.

We studied transmission of male vibrational signals between grapevine leaves that were separated by a gap of varying distance. In playback experiments (Figure 2), the mean substrate velocity progressively decreased with the distance (i.e. width of the gap) (Jonckheere test: J_0_ = 5.93, P<0.001). In contrast, the dominant frequency increased (J_0_ = 2.29, P = 0.011). Compared with the signal recorded from the lower leaf, at 0.5 cm gap distance the decrease in vibration velocity was on average of 91.6±7.1% and at 11 cm gap distance the velocity was further reduced of 7.3±5.6%. Values of velocity measured between 0.5–1 cm were over 0.001 mm/s, whereas from 2 cm gap the mean velocity was constantly lower.

### Test 3. The role of antennae in perception of vibrational signals

When ten pairs of intact males and females with surgically removed antennae were tested on the same leaf, all females responded to the MCS. When pairs were tested on two leaves not connected via the common substrate and separated by 5 cm gap, seven out of ten females responded. This result is identical to test 1, when leaves were separated by 5 cm gap and females had intact antennae.

## Discussion

Contrary to general belief, our findings demonstrate that the communication range of vibrational signals emitted by small insects is not limited to physically interconnected substrates. Production of low-frequency acoustic signals that are perceived by receivers as substrate-borne vibrations usually also results in emission of a low-intensity air-borne component [6], [20], [21]. Efficient radiation of acoustic sources in the air is possible only when emitter is bigger than 1/3 of the wavelength of the emitted sound [31], [32]. For an insect of the size of *S. titanus* (4–5 mm), the optimal frequency of air-borne sound would be above 10 kHz. The effective air-borne range of low frequency vibrational signals with dominant frequencies in the range between 80–300 Hz emitted by *S. titanus* is short and we never heard air-borne sounds during their calling. Nevertheless, while communication at distances larger than a few cm is mediated by vibrations of the substrate, at closer range the role of air-borne component cannot be excluded. At a range of a few cm, such signals may be detected by mechanosensory hairs [33] or the Johnston's organ in the antennae [22]. Our results show that in *S. titanus* mechanoreceptors in the antennae are not involved in detection of air-borne component of vibrational signals. Heteropteran insects possess hairs that may be used for detecting air-particle displacement [34] however, a systematic survey of sensilla on the leafhopper body is lacking.

Our measurements showed that vibrations are transmitted from one leaf to another even when they were separated by a gap of 11 cm and that females responded to males up to a gap width of 6 cm. From our results it was not possible to determine explicitly whether the vibrational signals were detected as air-borne sound or as substrate vibrations induced in the leaf. However, some observations, indicate the latter as the more probable hypothesis. In some cases male and female leafhoppers were not positioned within the gap between leaves, but on external sides of leaf laminae. In such situation two leaves would represent severe obstacle to any low intensity air-borne sounds. On the other hand, it has previously been shown that leaf vibrations are transmitted through the air beyond the boundary layer of the leaf and that air particle displacement triggered by leaf vibrations has the same temporal pattern as substrate vibrations [35]. The fact that in our experimental set-up we used two partly overlapping leaves with relatively large surface may also explain why in other studies in which only the tips of the leaves were in close proximity, concluded that vibrational communication was limited to a continuous substrate. Situations in which leaves are separated by a gap but partly overlapping probably represent a more natural case for insects that communicate in a dense vegetation habitat.

The maximum intensity of vibrational signals on a leaf without any contact with the vibrated leaf, measured directly as velocity at gap distances at which females were still responding, was in the velocity range between 10^−6^ and 10^−7^ m/sec at dominant frequencies between 220–250 Hz. These values translate to displacement values between 10^−9^ and 10^−10^ m. The lowest neurobiologically determined velocity threshold values for subgenual organs in various insect groups are all in the range between 10^−5^ and 10^−6^ m/sec (Heteroptera: [36], [37]; Neuroptera: [38]; Orthopteroids: [39]–[41]). However, in all these insects conversion of velocity threshold values into displacement values results in threshold values below 10^−9^ m. In particular, in another hemipteran insect, the southern green stink bug *Nezara viridula*, threshold values of receptor cells in the subgenual organ follow the line of equal displacement [36]. This suggests that, although displacements induced in a leaf by vibrational signals emitted on another leaf nearby are low, they are not below the threshold values of the subgenual organ. In leafhoppers nothing is known about vibration receptors in the legs [10]. However, it is likely that leafhoppers possess subgenual organs on all six legs. In insects this is the most sensitive organ to detect substrate vibrations and it was described also in closely related insect groups such as froghoppers (Cercopidae) and bugs (Heteroptera) [36], [42], [43]. Our measurements also revealed a significant increase in dominant frequency (from 200 to 250 Hz) when vibrational signals were transmitted through air from one leaf to another. It is interesting to note that resonant frequencies of sound-induced vibrations in bean leaves are in the frequency range between 190 and 290 Hz [44]. In the pentatomid bug *N. viridula*, for which bean is a preferred host plant, resonant frequencies correspond to best frequency sensitivity of one of the two cells in the subgenual organ [37]. We argue that transmission of vibrational signals from one leaf to another via air may be a common phenomenon. High receptor sensitivity, together with potential tuning of plant resonant frequencies with spectral properties of vibrational signals may enable the insect to extend the communication range beyond the limit of one plant.

In addition, our results suggest that the intensity of the perceived vibrational signals may have crucial effects on the leafhopper behavior. Mating duet followed by female location was observed only at the two shortest gaps, while call-fly behavior prevailed at longer distances. Although the role of shifts in dominant frequency cannot be excluded, the observed differences are small (between 20 and 40 Hz) in comparison with the 20 dB difference in intensity. When male and female were positioned on the same leaf at the beginning of our observations, MCS was immediately extended into a courtship phrase without the intermediate stage observed at other distances [29], [45]. It is conceivable that leafhoppers are able to compare the intensity of their own signals and perceived signals emitted by the duetting partner. Below a certain threshold the intensity may provide information that the female is not located on the same leaf as the male and that the male therefore needs to adjust the searching strategy accordingly. Since most studies on planthopper and leafhopper mating behavior have been conducted in short range situations, the information about patterns of long-range communication is lacking.

The call-fly behavior observed in males is usually associated with a strategy to increase effective signaling space [30], [46]. However, when the position of the source of low intensity female reply is unpredictable for the courting male, call-fly strategy may enable a faster localization of the leaf hosting the female. In addition, numerous changes of the position of the signaling male may reduce predation risk from eavesdropping predators like spiders [47].

In conclusion, we showed that the communication range of vibrational signals is not limited by substrate continuity and that in this situation antennal receptors are not essential in detection of vibrational mating signals. Moreover, our behavioral observations together with measurements of signal transmission between grapevine leaves suggest that behavioral responses of *S. titanus* may depend on the signal intensity.

## Materials and Methods

### Rearing of insects

*S. titanus* eggs originated from two-year-old grapevine (*Vitis vinifera*) canes collected from organic farms in Northern Italy (Povo, Trento, Italy). Egg hatching occurred in a climate chamber (24±1°C, 16L∶8D photoperiod, 75% R.H.). Nymphs were removed daily into rearing boxes, consisting of plastic beakers (height 10 cm; 5 cm i.d.) with a moistened grapevine leaf laid on top of a 1-cm-layer of technical agar solution (0.8%) that was replaced twice a week. At emergence, adults were separated by sex and age (day of emergence), and kept in the rearing boxes. All experiments were made with virgin, sexually mature males and females at least 8 days old [29].

### Terminology and recording of vibrational signals

In the current study we used terminology established by Mazzoni et al. [29].

The experiments were performed in an enclosed room of the Entomology Section (Pisa University) at 23±1°C from June to August, between 5 pm and 9 pm which is the peak in sexual activity in *S. titanus* [29]. The signals were recorded with a laser vibrometer (Ometron VQ-500-D-V, Brüel and Kjær Sound & Vibration A/S, Nærum, Denmark) and digitized with 48 kHz sample rate and 16-bit resolution, then stored directly onto a hard drive through Plug.n.DAQ (Roga Instruments, Waldalgesheim, Germany). Signal spectral analysis was performed by means of Pulse 14 (Brüel and Kjær Sound & Vibration A/S). Recorded signals were analyzed with a FFT window length of 400 points. The leafhopper behavior was recorded with a Canon MV1 miniDV camera. The communication between males and females was observed for 20 minutes or until the male reached the female.

### Test 1. Inter-plant communication

**Figure 3:**
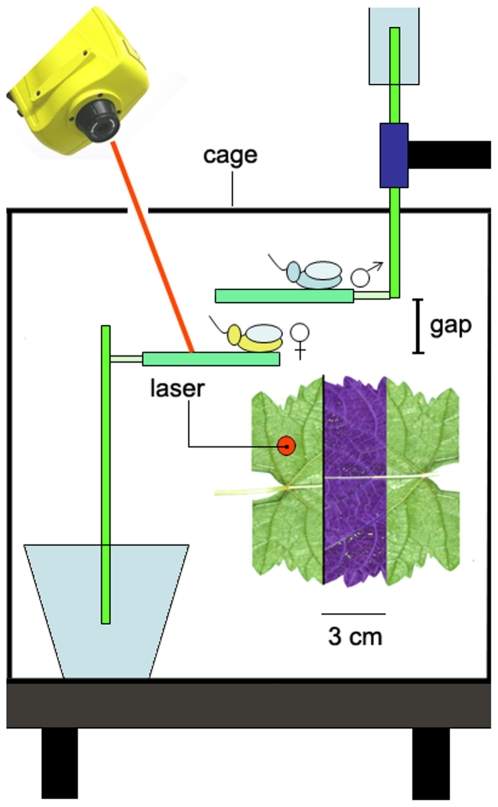
A schematic drawing of experimental setup. A male and a female were placed on leaves (surface 6×10 cm) of two separate grapevine cuttings. The bottom of the stem was put in a glass vial filled with water to prevent withering. One cutting was put on an anti-vibration table (Astel S.a.s., Ivrea, Italy). The second cutting was attached to a metal arm suspended from above – without any contact with the table - and positioned in parallel over half the surface of the lower leaf (as shown in the inset as viewed from above). The laser beam was focused on the lamina of the lower leaf with the female. To prevent the insects from escaping, recordings were made within a Plexiglas cylinder (50×30 cm), provided of two openings for the laser beam and the metal arm. Not drawn to the scale.

We placed a male and a female on leaves of two separate grapevine cuttings with one leaf (surface 6×10 cm) (see Figure 3). The gap width between the upper and lower leaf surface ranged from 0.5 cm to 7 cm. For each distance we recorded whether the female responded to the MCS emitted by male with the prompt emission of pulses. Then, we categorized and counted the male behavioral reactions to the female reply: (1) no reaction; (2) mating duet followed by call-fly; (3) mating duet with male search and location of the female.

### Test 2. Signal transmission

Transmission of MCS between grapevine leaves that were not connected by a common substrate was studied by playback of pre-recorded MCS. The spectral structure of *S. titanus* MCS is characterized by a series of several prominent frequency peaks in the range between 80 and 300 Hz and maximum substrate vibration velocity above 10^−2^ mm/s. We recorded MCS at a close range on the grapevine leaf with a laser vibrometer as described above, from three different males. Since variability between spectral parameters among males was negligible we used a single randomly chosen MCS (composed of 27 pulses). Five pairs of leaves were tested from different cuttings, in the same experimental set up of Figure 3, in absence of real insects and cage. The lower grapevine leaf was vibrated by a minishaker (Type 4810; Brüel and Kjær Sound & Vibration A/S) with a conical tip attached onto the leaf surface, 2 cm distant from the anterior border. The minishaker was driven from a computer via Adobe Audition 3.0 (Adobe Systems Incorporated). The amplitude of playback signal was adjusted to the natural emitted signal. The measurements were taken from the leaf lamina in two different randomly chosen points at least 2 cm distant from the border both of the lower and upper leaf by laser vibrometer. The gap between parallel leaf surfaces was 0.5, 1, 2, 3, 4, 5, 6, 7 and 11 cm. Spectral components and velocity of leaf vibration were analyzed along the distance by taking the average of nine randomly chosen recorded pulses from each distance and each leaf. To assess the velocity and frequency differences the Jonckheere test was performed [48].

### Test 3. The role of antennae in perception of vibrational signals

Females were put in a freezer (−25°C) for 30 seconds to cool them and prevent them from moving when placed under a stereomicroscope. Both antennae were cut off with microscissors. After ablation, females were kept separately in the rearing boxes for 24 hrs before they were used in experiments.

For the experiments, ten pairs consisting of intact males and of females whose antennae had been removed were first tested at close range on a single grapevine leaf to determine the female responsiveness after the ablation. In case of female response, they were subsequently tested on two leaves not connected via the common substrate and separated by a 5 cm gap as described above. The laser was focused on the leaf of the female.
